# Case of Human Infestation with *Dermanyssus gallinae* (Poultry Red Mite) from Swallows (Hirundinidae)

**DOI:** 10.3390/pathogens10030299

**Published:** 2021-03-04

**Authors:** Georgios Sioutas, Styliani Minoudi, Katerina Tiligada, Caterina Chliva, Alexandros Triantafyllidis, Elias Papadopoulos

**Affiliations:** 1 Laboratory of Parasitology and Parasitic Diseases, School of Veterinary Medicine, Faculty of Health Sciences, Aristotle University of Thessaloniki, 54124 Thessaloniki, Greece; Gsioutas@vet.auth.gr; 2 Department of Genetics, Development and Molecular Biology, School of Biology, Aristotle University of Thessaloniki, 54124 Thessaloniki, Greece; sminoudi@bio.auth.gr (S.M.); atriant@bio.auth.gr (A.T.); 3 Department of Pharmacology, Medical School, National and Kapodistrian University of Athens, 10679 Athens, Greece; aityliga@med.uoa.gr; 4 Allergy Unit “D. Kalogeromitros”, 2nd Department of Dermatology and Venereology, National and Kapodistrian University of Athens, 12462 Athens, Greece; cchliva@gmail.com; 5 Medical School, University General Hospital “ATTIKON”, 12462 Athens, Greece

**Keywords:** *Dermanyssus gallinae*, gamasoidosis, poultry red mite, swallows, COI gene, PCR, avian mite dermatitis, Greece

## Abstract

*Dermanyssus gallinae* (the poultry red mite, PRM) is an important ectoparasite in the laying hen industry. PRM can also infest humans, causing gamasoidosis, which is manifested as skin lesions characterized by rash and itching. Recently, there has been an increase in the reported number of human infestation cases with *D. gallinae*, mostly associated with the proliferation of pigeons in cities where they build their nests. The human form of the disease has not been linked to swallows (Hirundinidae) before. In this report, we describe an incident of human gamasoidosis linked to a nest of swallows built on the window ledge of an apartment in the island of Kefalonia, Greece. Mites were identified as *D. gallinae* using morphological keys and amplifying the Cytochrome C oxidase subunit I (COI) gene by PCR. Bayesian phylogenetic analysis and median-joining network supported the identification of three PRM haplogroups and the haplotype isolated from swallows was identical to three PRM sequences isolated from hens in Portugal. The patient was treated with topical corticosteroids, while the house was sprayed with deltamethrin. After one week, the mites disappeared and clinical symptoms subsided. The current study is the first report of human gamasoidosis from PRM found in swallows’ nest.

## 1. Introduction

The arthropod parasite *Dermanyssus gallinae* (De Geer 1778), also known as the poultry red mite (PRM), is one of the most important ectoparasites in the modern laying hen industry with a worldwide distribution [1]. Through its blood-sucking action, it is responsible for annual financial losses of about 231 million euros in Europe that are mainly attributed to the resulting reduced egg production and quality and increased morbidity and bird mortality due to anemia [2]. In addition, the PRM has been reported to carry or act as a vector of important zoonotic pathogenic microorganisms, including bacteria *Salmonella enteritidis* [3,4], *Erysipelothrix rhusiopathiae* [5], *Chlamydia psittaci* [6], *Escherichia coli* [7], *Pasteurella multocida, Coxiella burnetii*, and *Listeria monocytogenes* [8], and influenza type A virus [9]. However, for most pathogens, only isolation has been proven from PRM infesting hens. Vectorial competence remains to be demonstrated for some in order to clarify its medical significance in transmission to humans. In relation to urban cases of gamasoidosis, *Borrelia burgdorferi sensu lato* and *Coxiella burnetii* have also been isolated from *D. gallinae* [10]. Moreover, the DNA of *Bartonella quintana* was isolated from PRMs infesting a family house, whose members showed symptoms of trench fever [11].

The genus *Dermanyssus* includes at least 25 species [12]. Specifically, in the *D. gallinae* complex, at least 2 clades have been described that are organized in several genealogical series [13]. The different haplotypes that have been identified allow the genetic discrimination between the special lineage L1 of *D. gallinae* that infests mainly urban pigeons *(Columba livia domestica*) and other Columbiformes species and the *D. gallinae sensu stricto* that infests hens (*Gallus gallus domesticus*) and swallows (Hirundinidae) among others. Both cryptic species can infest humans, with *D. gallinae* L1 having more medical relevance in urban areas, where pigeons and doves are common, and *D. gallinae sensu stricto* usually on poultry farms where hens are housed [14,15,16]. *D. gallinae* infests more than 30 wild bird species but has no strict host specificity compared to other *Dermanyssus* species, which do not alternate between hosts as easily. The increasing incidences of PRM attacks beyond birds indicates expansion to other hosts [12,14]. Global warming, travelling, and increases in the human population, farms, and small animal densities are all thought to facilitate further host expansion [17].

During the day, *D. gallinae* hides in cracks, crevices, or nests away from light where birds cannot peck and eat them. Its life cycle is direct, and under ideal conditions of temperature (10–35 °C) and relative humidity (>70%), it is completed within seven days, causing the formation of large colonies and rapid increases in the population. Contrary to the larvae with six legs that do not engorge in blood, the nymphs and adults have eight legs and are hematophagous. Adults feed at night for less than 2 h every 2–3 days and turn red after engorging in blood. Females can reach a length of 1 mm and lay their eggs shortly after a meal. They survive up to eight months without feeding [1,18], but when the host birds are absent, as is the case of abandoned nests by the young pigeons, large PRM populations can forage short distances or relocate to a new food source in search of a blood meal, utilizing temperature stimuli, response to vibrations, and carbon dioxide to locate their host [19].

Most gamasoidosis cases are reported in late spring or early summer [20,21]. Workers at infested poultry houses are at a higher risk for gamasoidosis, described as an “occupational hazard” in their work field [22]. In the urban environment, gamasoidosis cases are associated with the proliferation of pigeons in cities where they build their nests on roofs, air-conditioning boxes, window sills, eaves, and air ducts [20,21,23]. Gamasoidosisis is mainly caused by *D. gallinae*, but other species, such as *Ornithonyssus sylviarum* (northern fowl mite), *O. bursa* (tropical fowl mite), and *D. avium* may also be causative agents [24]. The resulting PRM-associated dermatitis is a local or generalized non-characteristic skin reaction that may be misdiagnosed as scabies or pediculosis [25]. Skin lesions are usually flattened erythematous papules, intensely pruritic that may affect various parts of the body. Dermatoscopic criteria for the disease have not been described, but dermatoscopy may help rule out delusional parasitoses [26]. The clinical signs are self-limited and usually resolve abruptly, although symptomatic treatment with topical corticosteroids and local or oral antihistamines has been reported [25]. On the other hand, prevention is based on strict monitoring of the infestation site, on the removal of bird nests containing PRM, and on the cleaning and disinfection of the infested area with an appropriate acaricide [16,21].

To the authors’ knowledge, there are only limited reports in the available literature on *D. gallinae* in swallows (Hirundinidae) or their nests, as swallows are typically infested with *D. hirundinis*. In a report from France, *D. gallinae* constituted 16% of the *Dermanyssus* mites found in the swallows’ nests [12]. In a second report, *D. gallinae* was identified in 61 out of 161 (approx. 38%) barn swallows’ nests (*Hirundo rustica*) examined for mites in Iran [27]. In both reports, most nests were built near or right next to poultry houses, suggesting a migration of PRM from hens to swallows.

## 2. Case Presentation

### 2.1. Clinical Description

**Figure 1:**
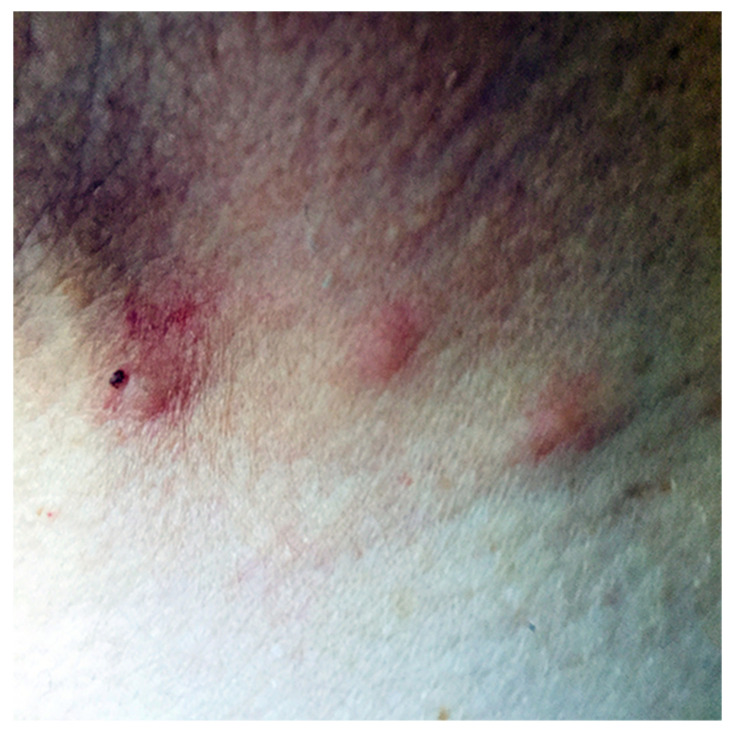
Skin lesions of gamasoidosis following human infestation with *Dermanyssus gallinae*. Excoriated erythematous papules and urticaria-like plaques characterized by rash and itch developed within minutes after the bites.

A gamasoidosis case was recently recorded in Argostoli, the capital of the Ionian island of Kefalonia, Greece. A 37-year-old male agronomist presented with scattered groups of pruritic, erythematous papules with a size of 3 mm and a central punctum in various parts of his body, including the upper and lower limbs. The lesions appeared suddenly in mid-May 2020, especially in the morning hours after waking up, and continued to develop for just over a month (Figure 1).

The patient visited the emergency department of the local public hospital. He reported that he had experienced more than 20 repeated bites and that local urticarial rash and edema developed within minutes after the bites. Apart from the cutaneous lesions, clinical signs and symptoms from other systems were not reported. There was no personal or family history of any allergic disease, no previous experience of a hypersensitivity reaction, no contact with animals in his daily life, and no pets in the apartment.

In the weeks prior to the onset of the symptoms, the patient had noticed swallows building their nest on the window sill of his privately owned second-floor apartment. A few days after the onset of the symptoms, the patient saw mites crawling in the room and on his body, whereas large mite numbers were present in the swallows’ nest. After contacting the Laboratory of Parasitology and Parasitic Diseases, School of Veterinary Medicine, Aristotle University of Thessaloniki, Greece, the patient was instructed to provide a sample of mites in vials containing ethanol (70% *v*/*v*) as a preservative, as well as to fill in a medical history questionnaire (Figure S1).

### 2.2. Morphological Identification of the Mites

**Figure 2:**
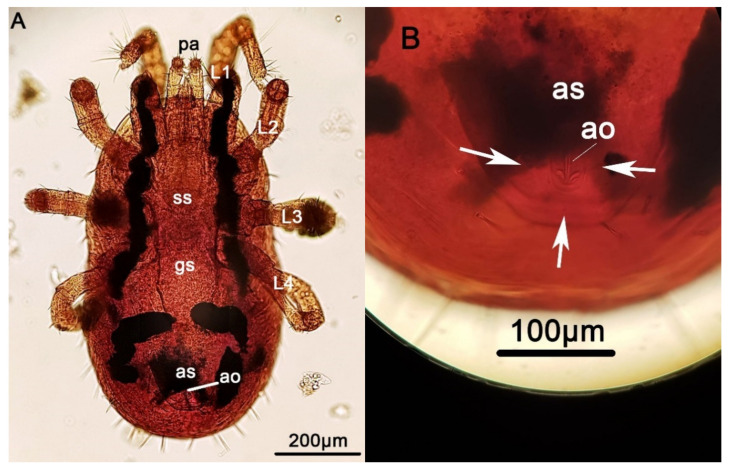
Ventral view of a female *Dermanyssus gallinae* mite under the light microscope at (**A**) 100× magnification and (**B**) 400× magnification, detail of the anal shield. Terminology based on keys described by Moss et al. [28,29] and Di Palma et al. [30]: as: anal shield; ao: anal opening; gs: genitoventral shield; ss: sternal shield; L1–4: leg pairs; pa: palp. The arrows pinpoint at the three anal setae.

The collected mites were mounted on slides using lactophenol as the mounting medium. After examination under the stereomicroscope (Olympus, Research Stereomicroscope System SZH10) and the light microscope (Olympus, CX21 Microscope) at 100× and 400× magnification using key morphological criteria [28,29,30], the mites were identified as *D. gallinae* (Figure 2). Further distinction of *D. gallinae* between the two major linage clades requires DNA analysis [13] and it was not possible to be performed based solely on the morphology. In the reported case, amplification of the Cytochrome C oxidase subunit I (COI) gene by the polymerase chain reaction (PCR) was used as a marker as proposed by Chu et al. [31].

### 2.3. Molecular Identification and Phylogenetic Analysis of the Mites

Total genomic DNA was extracted from 5 individuals mites using QIAamp DNA mini kit Extraction Kit (Qiagen, Hilden Germany), with some modifications. Mites were homogenized by cutting open their whole body with a sterile Agani^TM^ 21G × 1 ½” (0.8 × 38 mm) needle. The proteinase K digestion step was performed overnight at 56 °C, and the volumes of all the reagents were modified as follows: 90 μL of ATL buffer, 10 μL of proteinase K, 100 μL of Al buffer, 100 μL of ethanol, 250 μL of AW1 buffer, and 350 μL of AW2 buffer. Extracted DNA was eluted in 50 μL of AE buffer and stored at −20 °C until PCR amplification.

A fragment of 681 bp of the COI gene (Cytochrome C oxidase subunit I) of mitochondrial DNA (mtDNA) was amplified using primers COI1Fyuw114 and COI1Ryuw114 [32]. The total volume of PCR reaction was 30 μL consisting of 100 ng of genomic DNA as a template, 0.05 units of Qiagen Taq polymerase (Qiagen, Hilden Germany), 2 mM deoxynucleotide triphosphates (dNTPs), 0.3 μL of each primer (100 μΜ), 2.5 mM MgCl_2_ (Qiagen, Hilden Germany), and 3 μL of 10× Reaction Buffer (Qiagen, Hilden Germany). A Takara PCR Thermal Cycler (Takara BIO INC, Japan) was used and amplification conditions were the following: initial denaturation at 95 °C for 5 min, 38 cycles of strand denaturation at 95 °C for 30 s, annealing at 55 °C for 45 s, and primer extension at 72 °C for 30 s, before a final elongation of 5 min at 72 °C. Amplification success was assessed by electrophoresis of the PCR products in 1.5% (m/v) agarose gels (AppliChem, Darmstadt, Germany) with subsequent visualization under ultraviolet light. All PCR products were sent to the company Genewiz (Takeley, Essex, UK) for purification and sequencing.

Sequences were checked and analyzed using Geneious version 10.2.6 [33]. Sequence data from the present study were combined with 298 COI sequences of *D. gallinae* isolated from hens (195 sequences: LR812140, LR812284-LR812477 from [32], 103 sequences: LC029457-LC029557 from [31]), 2 sequences of *D. gallinae* isolated from swallows (AM921866-7 from [13]), 3 sequences isolated from pigeons (AM921859, AM921859 from [13], LT714694 from [16]), and 8 sequences of *D. hirundinis* isolated from swallows (FM179366, FM179369, AM921872 from [13], FM208740-1, FM208726-8 from [12]). The overlapping region was 457 bp.

All the sequences were aligned using Clustal W [34]. The different haplotypes were found by using the program DNAsp v6 [35]. The best-fit nucleotide substitution model for this dataset was determined using MEGA X [36]. The HKY + G model was used for Bayesian phylogeny analysis, carried out with Beast 1.10.4 [37]. The Bayesian tree was constructed using a strict clock model and a coalescent tree prior. The analysis was run for 10^8^ Markov Chain Monte Carlo (MCMC) generations, sampled every 10^4^ generations. Convergence of chains was visualized using Tracer 1.7 [38] discarding the first 20% of trees as burn-in. Effective Sample Size (ESS) values for all parameters were larger than the threshold value of 200 identified by Tracer v.1.7. The trees produced by Beast were then summarized in TreeAnnotator 1.10.4 and visualized in FigTree 1.4.3 [39].

Additionally, a median-joining network [40] was constructed using the software Network 10.2.0 and the frequencies of the sequences (Fluxus Technology) assuming equal weights for all mutations and setting the genetic distance parameter to zero in order to restrict the choice of feasible links in the final network.

DNA sequencing was successful in four out of five samples. We obtained a single 529-bp DNA sequence (haplotype) from all four mite individuals. The haplotype we recovered was also the same as three identical sequences from mites isolated from hens in Portugal (Accession numbers: LR812310-2) [32]. The sequence identified in the present study was deposited in GenBank with Accession number: MW542575.

**Figure 3:**
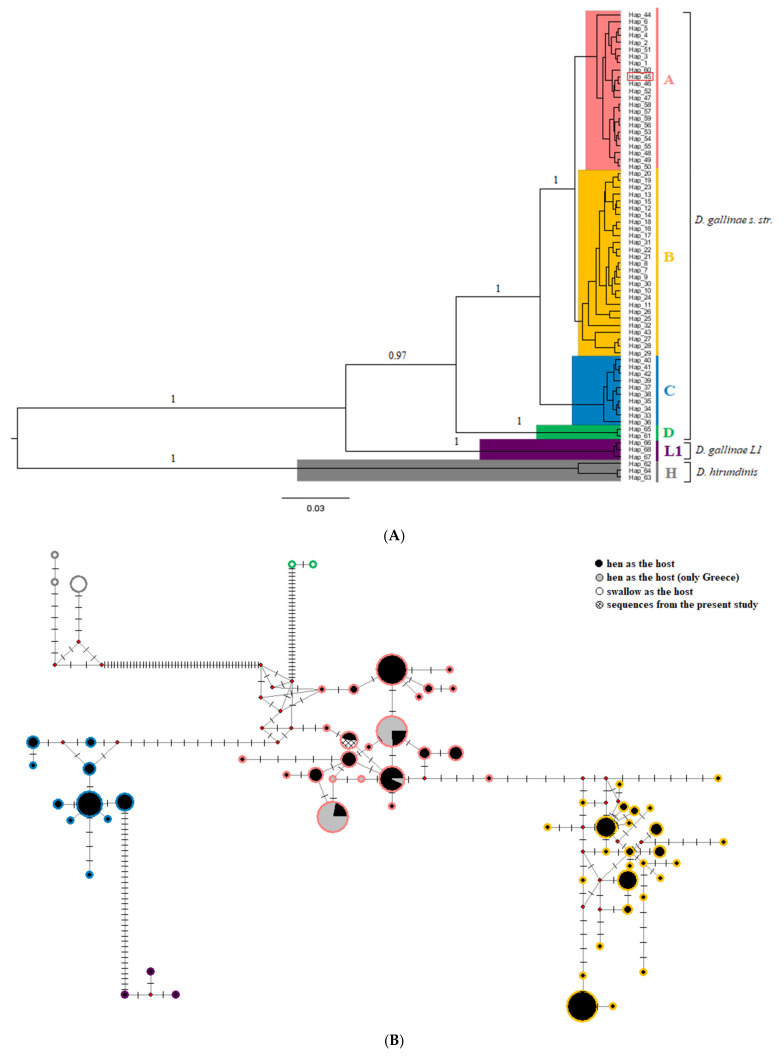
Haplogroups A (red), B (yellow), C (teal), and D (green) constitute *Dermanyssus gallinae sensu stricto* cryptic species and haplogroup L1 (purple), *Dermanyssus gallinae* L1 cryptic species. Haplogroup H (gray) represents *Dermanyssus hirundinis*. Mites belonging to haplogroup A (red), B (yellow), and C (teal) have been isolated from hens, haplogroup D (green), and H (gray) from swallows, and haplogroup L1 (purple) from pigeons. The single haplotype identified in the current study (haplotype 45) fits in haplogroup A (red) and is highlighted accordingly. (**A**) Bayesian phylogenetic tree of mites’ haplotypes. Posterior probabilities are shown to the main clades. (**B**) Median-joining network of mtDNA mites’ haplotypes. The proportional size of nodes indicates the frequency of haplotypes. The colored line of circles corresponds to the colored haplogroups in the phylogenetic tree (Figure 3A). Small red dots represent inferred haplotypes.

The Bayesian phylogenetic analysis (Figure 3A) and the median-joining network (Figure 3B) supported the identification of three haplogroups as regards clustering haplotypes coming from mites *D. gallinae sensu stricto* isolated from hens. Haplogroup A contains 23 haplotypes from 10 countries, including the unique haplotype identified in the present study. All mite haplotypes (*n* = 5) previously identified in Greece from Karp-Tatham et al. [32], corresponding to 61 sequences, are also grouped in this cluster. Haplogroup B includes 27 haplotypes from 8 different countries. Finally, haplogroup C includes 10 haplotypes coming from 7 countries. The haplotypes of *D. gallinae* isolated from swallows clustered in one distinct haplogroup (D), which was separated by several mutational steps (*n* = 32) by the other haplogroups of the species. The special lineage L1 contains haplotypes from *D. gallinae* mites isolated only from pigeons. The haplotypes of *D. hirundinis* construct one more distinct haplogroup (H). According to the median-joining network, the two species (*D. gallinae sensu stricto* and *D. hirundinidis*) are separated by 55 mutational steps. All accession numbers and countries of origin for each haplotype used in the current study are presented in Table S1.

### 2.4. Treatment and Control of the Mite Infestation

After visiting the emergency department of the local public hospital, the patient was prescribed 0.1% w/w cream methylprednisolone aceponate as topical corticosteroid treatment. Following the identification of the causative agent, thorough cleaning and vacuuming of the apartment was recommended, as well as thorough spraying for a few days with a commercial insecticide formulation, containing 2.5% deltamethrin (Deltamethrin 2.5 WP, FARMA—CHEM SA, Thessaloniki, Greece; 4–6 g/L H_2_O, sprayed onto 8–12 m^2^ surface area; application only when mites are seen according to the manufacturer’s instructions). The choice of deltamethrin was based on its previous reported success in combating *D. gallinae* infestations and on experts’ recommendations [16,41]. These measures were applied together with the removal from the skin by bathing of any mites crawling on the patient and treatment with topical corticosteroids. After about 1 week, no mites were detected in the apartment and the clinical symptoms subsided. During the following month, no mites were spotted in the apartment and no symptom recurrence was reported by the patient. In the meantime, the young swallows had fledged their nest and did not return, so the owner could finally spray the swallows’ nest with deltamethrin to eliminate any remaining mites.

## 3. Discussion

Previous cases of human gamasoidosis have been linked to *D. gallinae* L1 mites infesting pigeons [16,42] and *D. gallinae sensu stricto* mites from hens [43]. It is uncommon for swallows to be infested with the PRM, since they are usually infested with *D. hirundinis* [12]. Moreover, this is the first reported human gamasoidosis case caused by *D. gallinae* due to an infested swallows’ nest.

In Greece, swallows are migratory birds that return to their breeding grounds from Africa in spring (March–April) and form pairs. Their breeding season lasts from March to June, and the pair builds their nest using mud pellets, feathers, grass, and other materials forming a cup. Usually, the nest is built next to human-made structures and the pair lives close to humans. The female typically lays 3–6 eggs that hatch in 15–20 days. The chicks fledge their nest when they are around 3 weeks old. At the end of August, before autumn begins, the swallows migrate back to the African continent due to the warmer climate there and the scarcity of insects at that time in the European continent [44]. Based on the patient’s description of the swallows’ exterior features, the endemic swallow species observed in the region [45], and on the nest site (window sill), it can be deduced that the most probable swallow species was *Delichon urbicum*. In the two previous reports of *D. gallinae* infesting swallows’ nest, the swallows’ species were identified as *Hirundo rustica* in Iran [27] and *Delichon urbicum* in France [12].

The *D. gallinae* haplotype isolated in the present study fits in haplogroup A among other PRM haplotypes identified in Greece and belongs to *D. gallinae sensu stricto* cryptic species. However, it is quite distant (32 mutational steps) from the two *D. gallinae* haplotypes that have been previously isolated from swallows in France (haplogroup D). This genetic distance can be explained by the fact that swallows are rare hosts for *D. gallinae* and only two other haplotypes from swallows have been studied. The close similarity between the haplotype identified in this study and other PRM haplotypes found in Greek hens suggests that the swallows may have acquired the mites after being in contact with hen facilities. Certain strains of *D. gallinae* mites can be transferred between hosts easily and continue to feed and reproduce on the new host without any problem [12].

The haplotype isolated from swallows in Kefalonia is identical to the haplotypes identified in PRMs isolated from hens in Portugal. These two places are almost 2500 km apart. Swallows have been shown to migrate to Portugal from Africa in February–June and travel large distances until they arrive at their breeding ground [46]. Since *D. gallinae* do not live on the hosts, but rather on their nests [27], it is unlikely that the mites identified in this study survived on the swallows at such large distance so as to be transferred from Portugal to Greece. One possible explanation would be that laying hens in Greece and Portugal might have been infested with the same *D. gallinae* haplotypes which was then transferred through the international trade of contaminated hens and equipment [15,17]. Furthermore, when building their new nests swallows tend to use materials from old abandoned nests [44] that, in this case, could possibly have been infested with mites.

In addition, swallow nests are sometimes found outside poultry facilities [12,27]. As PRM prevalence in northern Greek laying hen farms has been recently reported to be 100% [47] and, the situation is likely to be similar in hen houses in Kefalonia, it is highly probable that the swallows in Kefalonia acquired the *D. gallinae* mites from a hen house when scavenging for nest materials.

Finally, this case further illustrates the need to consider gamasoidosis in the differential diagnosis of pruritic dermatitis with erythematous papules, excoriations, and urticaria-like plaques, even when they occur in patients living in a built environment in the absence of any poultry housing facilities. In urban areas, aside from sparrows, starlings and pigeons, nesting or roosting of migratory birds, such as swallows, may be a source of concern in human medicine and one health approaches.

## 4. Conclusions

Gamasoidosis remains an underdiagnosed pathological entity, often overlooked despite the increasing number of reported cases, especially in urban areas [25,26]. Aside from the hens and pigeons that are the typical hosts for *D. gallinae* in egg-laying farms and the urban environment, respectively, this case demonstrates that one should consider swallows (*Hirundinidae*) as a potential host of this ectoparasite. Future research should focus on isolating *D. gallinae* mites from laying hen farms in Kefalonia and compare their haplotypes with the one identified from the swallows. Consequently, homeowners in breeding areas should be warned to be especially careful when they see normally harmless swallow nests on or around their homes, which may increase the risk of infestation with PRM and therefore spread avian mite dermatitis, particularly in late spring and early summer.

## Data Availability

Publicly available datasets were analyzed in this study. This data can be found here: https://www.ncbi.nlm.nih.gov/genbank/ (accessed on 5 January 2021).

## Supplementary Materials

The following are available online at https://www.mdpi.com/2076-0817/10/3/299/s1, Figure S1: Medical history questionnaire. Table S1: Accession numbers and countries of origin for each haplotype.
