# Supplementary material for: Case of Human Infestation with Dermanyssus gallinae (Poultry Red Mite) from Swallows (Hirundinidae)
Source: Pathogens. 2021 Mar 4;10(3):299. doi: 10.3390/pathogens10030299 (PMC8001604; doi:10.3390/pathogens10030299)
Supplement: Supplementary file 1 [file pathogens-10-00299-s001.zip › pathogens-1127811-supplementary materials/pathogens-1127811-supplementary Figure S1.pdf]

ΕΡΩΤΗΜΑΤΟΛΟΓΙΟ *Dermanyssus gallinae*

ΗΜΕΡΟΜΗΝΙΑ ΓΕΝΝΗΣΗΣ: 14/01/1983 (X) άρρεν ◯ θήλυ

ΕΠΑΓΓΕΛΜΑ: ΓΕΩΠΟΝΟΣ Ζ.Π.

ΠΕΡΙΟΧΗ (ΝΟΜΟΣ/ΠΟΛΗ/ΔΗΜΟΣ): ΚΕΦΑΛΛΗΝΙΑΣ - ΑΡΓΟΣΤΟΛΙ

ΗΜΕΡΟΜΗΝΙΑ ΣΥΜΠΗΡΩΣΗΣ ΤΟΥ ΕΡΩΤΗΜΑΤΟΛΟΓΙΟΥ: 08/ 11 /2020

1. **Ποιά περίοδο δεχθήκατε τσιμπήματα (μήνας - έτος);**

   Από 15\05\2020 έως 20\06\2020

2. **Πόσα τσιμπήματα δεχθήκατε;**

   ◯ μηδέν ◯ 1-5 ◯ 6-10 ◯ 10-20 (X) >20

3. **Σε ποιά σημεία δεχθήκατε τσιμπήματα;**

   ◯ Κεφάλι (X) Κορμός (X) Άνω άκρα (X) Κάτω άκρα

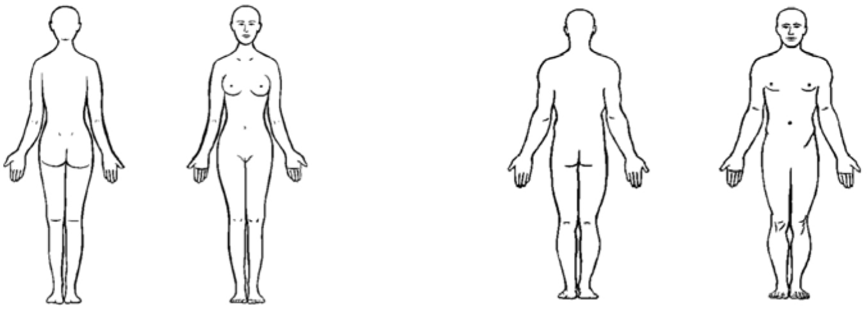

   (σημειώστε)

4. **Τι αντίδραση/συμπτώματα παρουσιάσατε μετά το τσίμπημα;**

   ◯ καμία/κανένα

   ◯ τοπική αντίδραση (<10cm)

   ◯ μεγάλη τοπική αντίδραση (>10cm)

   (X) κνίδωση/αγγειοοίδημα

   ◯ συστηματική αντίδραση [◯ δέρμα ◯ αναπνευστικό ◯ γαστρεντερικό ◯ καρδιαγγειακό]

   ◯ επιπεφυκίτιδα ◯ ρινίτιδα ◯ άσθμα ◯ βήχας ◯ δύσπνοια ◯ συριγμός ◯ βράγχος φωνής

   κνησμός ◯ παλαμών ◯ πελμάτων

   ◯ ναυτία ◯ έμετος ◯ διάρροια ◯ κοιλιακό άλγος

   ◯ ζάλη ◯ απώλεια αισθήσεων

   ◯ άλλο (περιγράψτε) ....

5. **Σε πόσο χρονικό διάστημα (min/h) μετά το τσίμπημα εμφανίστηκε η αντίδραση/τα συμπτώματα;**

   ΣΕ ΛΙΓΑ ΛΕΠΤΑ

6. **Επισκεφτήκατε τα επείγοντα λόγω της αντίδρασης από το τσίμπημα;**

   ◯ όχι (X) ναι ποιά ήταν η αντιμετώπιση (φάρμακα/εισαγωγή/κλπ); κορτιζονούχα αλοιφή

7. **Έκτοτε έχετε υποστεί νέο τσίμπημα;**

☒ όχι ○ ναι πότε (ημερομηνία) ....

παρουσιάσατε αντίδραση μετά από αυτό το τσίμπημα; ○ όχι ○ ναι

8. Έχετε ιστορικό αλλεργικής ρινίτιδος/επιπεφυκίτιδος (καταρροή, πταρμούς, μπούκωμα, κνησμό σε μύτη/μάτια, δακρύρροια);

☒ όχι ○ ναι [○ εποχιακά τους μήνες .... ○ σχεδόν όλο τον χρόνο]

9. Έχετε ιστορικό

άσθματος (βήχας, βράσιμο, δύσπνοια, βάρος στο στήθος)

☒ όχι ○ ναι [○ δεν έχει ○ έχει γίνει διάγνωση από γιατρό]

○ δεν λαμβάνω ○ λαμβάνω αντιασθματική αγωγή ○ περιστασιακά ○ συνεχώς]

ατοπικής δερματίτιδας (ξηροδερμία, έκζεμα)

☒ όχι ○ ναι [○ στην παιδική ηλικία ○ στην ενήλικη ζωή]

αντίδρασης μετά από τσίμπημα μέλισσας/σφήκας

☒ όχι ○ ναι προσδιορίστε .... πότε (ημερομηνία) ....

αλλεργίας/υπερευαισθησίας σε τροφή;

☒ όχι ○ ναι προσδιορίστε τροφή ....

Συμπτώματα ○ κνησμός σε στόμα, χείλη, φάρυγγα

○ συστηματική αντίδραση

αλλεργίας/υπερευαισθησίας σε φάρμακα

☒ όχι ○ ναι προσδιορίστε φάρμακο(α) ....

10. Παρουσιάζετε κνησμό, βήχα, ρινίτιδα κατά την χρήση προϊόντων από λάστιχο (γάντια, προφυλακτικά, μπαλόνια, κ.ά.);

☒ όχι ○ ναι προσδιορίστε σύμπτωμα ....

○ άμεσα ○ μετά από ………………….ώρες

11. Έχετε παρουσιάσει επεισόδια με εξάνθημα ή πρήξιμο σε χείλη, βλέφαρα, κ.ά.;

☒ όχι ○ ναι προσδιορίστε ....

12. Έχετε συγγενή 1ου βαθμού με ιστορικό αλλεργίας/ατοπίας;

☒ όχι ○ ναι προσδιορίστε ....

13. Λαμβάνετε κάποια φάρμακα;

☒ όχι ○ ναι προσδιορίστε (σκεύασμα) ....

14. Έχετε κάποιο πρόβλημα υγείας;

☒ όχι ○ ναι προσδιορίστε ....
