# Supplementary material for: Case of Human Infestation with Dermanyssus gallinae (Poultry Red Mite) from Swallows (Hirundinidae)
Source: Pathogens. 2021 Mar 4;10(3):299. doi: 10.3390/pathogens10030299 (PMC8001604; doi:10.3390/pathogens10030299)
Supplement: Supplementary file 1 [file pathogens-10-00299-s001.zip › pathogens-1127811-supplementary materials/pathogens-1127811-supplementary Table S1.docx]

**Table S1**: Accession numbers and countries of origin for each haplotype. The haplotype which includes sequences obtained in the present study is highlighted. The frequency for each country is presents in parenthesis.

| **Haplotype** | **Accession numbers** | **Countries of origin** |
| --- | --- | --- |
| Hap_1 | LR812290, LR812289, LR812477, LR812377, LR812376, LR812374 | Albania (2), UK (4) |
| Hap_2 | LR812445, LR812444, LR812446, LR812426, LR812425, LR812424, LR812423, LR812422, LR812421, LR812443, LR812442, LR812441, LR812440, LR812439, LR812438, LR812437, LR812436, LR812435, LR812419, LR812401, LR812400, LR812385, LR812384, LR812381, LR812380, LR812379, LR812367 | Greece (21), Netherlands (1), UK (5) |
| Hap_3 | LR812420 | Greece (1) |
| Hap_4 | LR812351, LR812350, LR812348, LR812347, LR812349 | Turkey (5) |
| Hap_5 | LR812352 | Turkey (1) |
| Hap_6 | LR812344 | Romania (1) |
| Hap_7 | LR812362, LR812337, LR812363, LR812471, LR812336, LR812388, LC029508, LC029516, LC029538, LC029495 | Belgium (2), Japan (4), Netherlands (2), UK (2) |
| Hap_8 | LR812361 | Netherlands (1) |
| Hap_9 | LR812360 | Netherlands (1) |
| Hap_10 | LR812316 | Czech Republic (1) |
| Hap_11 | LR812322 | Czech Republic (1) |
| Hap_12 | LR812470 | UK (1) |
| Hap_13 | LR812338 | Belgium (1) |
| Hap_14 | LR812320 | Czech Republic (1) |
| Hap_15 | LR812368 | Netherlands (1) |
| Hap_16 | LR812357 | Slovenia (1) |
| Hap_17 | LR812355, LR812353, LR812358 | Slovenia (3) |
| Hap_18 | LR812318, LR812319 | Czech Republic (2) |
| Hap_19 | LR812463, LR812462, LR812464, LR812465, LR812466, LR812469, LR812387, LR812386, LR812382 | UK (9) |
| Hap_20 | LR812468, LR812467 | UK (2) |
| Hap_21 | LR812364 | Netherlands (1) |
| Hap_22 | LR812288 | Albania (1) |
| Hap_23 | LR812389 | UK (1) |
| Hap_24 | LR812321 | Czech Republic (1) |
| Hap_25 | LR812326, LR812323, LR812324, LR812325 | Denmark (4) |
| Hap_26 | LR812356 | Slovenia (1) |
| Hap_27 | LR812331, LR812330, LR812329, LR812328, LR812327, LR812315, LR812314, LR812313, LC029498, LC029457, LC029459, LC029485, LC029557, LC029490, LC029556, LC029506, LC029554, LC029545, LC029541, LC029532, LC029509, LC029510, LC029514, LC029527, LC029546, LC029486, LC029475, LC029484 | Czech Republic (3), Denmark (5), Japan (20) |
| Hap_28 | LC029539 | Japan (1) |
| Hap_29 | LC029552 | Japan (1) |
| Hap_30 | LC029555, LC029530 | Japan (2) |
| Hap_31 | LC029543, LC029461 | Japan (2) |
| Hap_32 | LR812359 | Slovenia (1) |
| Hap_33 | LR812302, LR812297 | France (2) |
| Hap_34 | LR812295 | Croatia (1) |
| Hap_35 | LR812293, LR812298, LR812459, LR812457, LR812299, LR812456, LR812454, LR812453, LR812300, LR812365, LR812294, LR812366, LR812460, LR812296, LR812301 | Croatia (3), France (4), Italy (6), Netherlands (2) |
| Hap_36 | LR812461 | Italy (1) |
| Hap_37 | LR812354 | Slovenia (1) |
| Hap_38 | LR812292, LR812140, LR812286, LR812291, LR812287, LR812285, LR812284 | Albania (7) |
| Hap_39 | LR812458, LR812307, LR812455 | Italy (2), Portugal (1) |
| Hap_40 | LR812309, LR812308 | Portugal (2) |
| Hap_41 | LR812304, LR812303, LR812305 | Portugal (3) |
| Hap_42 | LR812306 | Portugal (1) |
| Hap_43 | LR812335 | Belgium (1) |
| Hap_44 | LR812334 | Belgium (1) |
| **Hap_45** | LR812312, LR812311, LR812310, **MW542575** | **Greece** (4), Portugal (3) |
| Hap_46 | LR812317 | Czech Republic (1) |
| Hap_47 | LR812399, LR812378, LR812375, LC029551, LC029550, LC029549, LC029548, LC029496, LC029497, LC029547, LC029526, LC029519, LC029517, LC029518 | Greece (1), Japan (11), UK (2) |
| Hap_48 | LR812343, LR812342, LR812452, LR812451, LR812450, LR812449, LR812448, LR812447, LR812433, LR812418, LR812432, LR812431, LR812430, LR812429, LR812428, LR812427, LR812417, LR812416, LR812415, LR812414, LR812413, LR812412, LR812411, LR812410, LR812409, LR812408, LR812407, LR812406, LR812405, LR812404, LR812403, LR812402, LR812398, LR812397, LR812396, LR812395, LR812394, LR812393, LR812392, LR812391, LR812341, LR812340, LR812373, LR812372, LR812371, LR812370, LR812369, LR812346, LR812345, LR812390 | Greece (38), Romania (6), UK (6) |
| Hap_49 | LR812475, LR812474, LR812472, LR812473, LR812476 | UK (5) |
| Hap_50 | LR812333, LR812332, LR812339 | Belgium (3) |
| Hap_51 | LR812383 | UK (1) |
| Hap_52 | LR812434 | Greece (1) |
| Hap_53 | LC029559, LC029467, LC029500, LC029482, LC029504, LC029463, LC029466, LC029501, LC029470, LC029499, LC029471, LC029474, LC029487, LC029489, LC029493, LC029536, LC029542, LC029529, LC029507, LC029478, LC029460, LC029528, LC029464, LC029468, LC029469, LC029472, LC029473, LC029476, LC029494, LC029477, LC029479, LC029480, LC029492, LC029481, LC029483, LC029488, LC029544, LC029531, LC029491, LC029524, LC029505, LC029523, LC029537, LC029512, LC029513, LC029521, LC029515, LC029535, LC029533, LC029534, LC029558, LC029511, LC029522 | Japan (53) |
| Hap_54 | LC029458 | Japan (1) |
| Hap_55 | LC029465 | Japan (1) |
| Hap_56 | LC029502 | Japan (1) |
| Hap_57 | LC029553 | Japan (1) |
| Hap_58 | LC029503, LC029540 | Japan (2) |
| Hap_59 | LC029520, LC029462 | Japan (2) |
| Hap_60 | LC029525 | Japan (1) |
| Hap_61 | AM921866 | France (1) |
| Hap_62 | FM208741, FM179369, AM921872, FM208728, FM208727, FM208726 | France (6) |
| Hap_63 | FM208740 | France (1) |
| Hap_64 | FM179366 | France (1) |
| Hap_65 | AM921867 | France (1) |
| Hap_66 | AM921861 | France (1) |
| Hap_67 | AM921859 | France (1) |
| Hap_68 | LT714694 | Italy (1) |
